# Non-linear dielectric spectroscopy of microbiological suspensions

**DOI:** 10.1186/1475-925X-8-19

**Published:** 2009-09-22

**Authors:** Ernesto F Treo, Carmelo J Felice

**Affiliations:** 1 Laboratorio de Medios e Interfases, Departamento de Bioingeniería; Facultad de Ciencias Exactas y Tecnología (FACET), Universidad Nacional de Tucumán (UNT), CC327, Correo Central, CP4000, San Miguel de Tucumán, Tucumán, Argentina; 2 Instituto Superior de Investigaciones Biológicas (INSIBIO), Consejo Nacional de Investigaciones Científicas y Técnicas (CONICET), Argentina

## Abstract

**Background:**

Non-linear dielectric spectroscopy (NLDS) of microorganism was characterized by the generation of harmonics in the polarization current when a microorganism suspension was exposed to a sinusoidal electric field. The biological nonlinear response initially described was not well verified by other authors and the results were susceptible to ambiguous interpretation. In this paper NLDS was performed to yeast suspension in tripolar and tetrapolar configuration with a recently developed analyzer.

**Methods:**

Tripolar analysis was carried out by applying sinusoidal voltages up to 1 V at the electrode interface. Tetrapolar analysis was carried on with sinusoidal field strengths from 0.1 V cm^-1 ^to 70 V cm^-1^. Both analyses were performed within a frequency range from 1 Hz through 100 Hz. The harmonic amplitudes were Fourier-analyzed and expressed in dB. The third harmonic, as reported previously, was investigated. Statistical analysis (ANOVA) was used to test the effect of inhibitor an activator of the plasma membrane enzyme in the measured response.

**Results:**

No significant non-linearities were observed in tetrapolar analysis, and no observable changes occurred when inhibitor and activator were added to the suspension. Statistical analysis confirmed these results.

When a pure sinus voltage was applied to an electrode-yeast suspension interface, variations higher than 25 dB for the 3rd harmonic were observed. Variation higher than 20 dB in the 3rd harmonics has also been found when adding an inhibitor or activator of the membrane-bounded enzymes. These variations did not occur when the suspension was boiled.

**Discussion:**

The lack of result in tetrapolar cells suggest that there is no, if any, harmonic generation in microbiological bulk suspension. The non-linear response observed was originated in the electrode-electrolyte interface. The frequency and voltage windows observed in previous tetrapolar analysis were repeated in the tripolar measurements, but maximum were not observed at the same values.

**Conclusion:**

Contrary to previous assertions, no repeatable dielectric non-linearity was exhibited in the bulk suspensions tested under the field and frequency condition reported with this recently designed analyzer. Indeed, interface related harmonics were observed and monitored during biochemical stimuli. The changes were coherent with the expected biological response.

## Background

An enzyme catalytic process is a cyclic reaction that responds to a periodic driving force with which the enzyme can interact. As a result of this interaction, the enzyme will oscillate between its different conformational states. For carrier enzymes, it has been assumed that this driving force is the free energy obtained from ATP hydrolysis. However, these enzymes can also couple energy conversion from transmembrane electric fields into an electrochemical gradient away from its equilibrium [1,2]. Membrane-bounded enzymes, as an electrochemical cyclic time-dependent processes, may appear as non-linear systems when exposed to a sinusoidal electric field [3]. This non-linear phenomenon was anticipated by simulations and analytical solutions [1,4-8].

Non-linear behavior of microbiological suspensions was tested with an externally applied sinusoidal electric field by the group of Woodward and co-workers [9-11]. Such non-linear system should generate harmonics on the polarization current and they can be measured with Fourier analysis [12]. Based on this analysis, Woodward designed a nonlinear spectrometer to evaluate some biological samples, mostly *Saccharomyces cerevisiae *suspensions [9]. They applied a sinusoidal voltage signal to a tetrapolar cell through the outer electrodes, they registered the voltage drop between the inner electrodes, and they analyzed it with Fourier series. The power spectrum obtained revealed the presence of harmonic components not included in the externally applied signal. However, each outer electrode generated an electrode-electrolyte interface (EEI) when contacted to the suspension. Within the voltage range evaluated, each EEI may be regarded as non-linear systems. The entire tetrapolar cell can be considered as a three-stage system. Two of these systems are certainly non-linear (the EEI), whereas the third one (the yeast suspension) is suspected to be so. In this scenario, the analysis of the signal measured in the inner electrodes reveals information about the biological medium and both EEI. If the measurements are carried out only in one culture cell containing yeast, there is no way to separate the EEI non-linearity from the biological one. To overcome the EEI interference, Woodward proposed to subtract the power spectrum of a similar cell with supernatant, from the power spectrum of the cell containing yeasts. This difference was attributed to the non-linear behavior of the yeasts in the suspension. The most representative finding was a prominent difference close to -25 dB of the third harmonic when the input signal was a sinusoidal electric field of ± 2 V cm^-1 ^and 20 Hz.

Later, Hutchings and co-workers, with different instrumentation, reported the absence of the non-linear behavior [13] suggesting the interfacial origin of the non-linearity [14] reported by Woodward. However, the arrangement proposed by Woodward was tested with other microorganisms [15,16]. In any case, the consistency of results obtained revealed a biological contribution to the non-linear phenomenon [17].

Since then some improvements were made to the instrumentation and to the data analysis [18,19] and several solutions were tried to avoid the unstable behavior of the EEIs [20], but there was not a great deal of advance in this promissory technique. Similar results were obtained with a radical different technology when Nawarathna, Miller and co-workers used both bipolar and tetrapolar analyzers. The former was depicted with two gold electrode and the current was sensed with a magnetic device [21], and the latter was a four gold electrodes arrangement, similar to Woodward's [22]. They could partially reproduce Woodward's result using the bipolar configuration [23,24]. Certainly, the non-linear nature of electrode polarization was recognized as a significant obstacle [25] by other researchers.

We believe that in the cited literature the instrumentation has not properly been designed to eliminate the EEI contribution, and the interfaces involved have not adequately been taken into account. None of these authors have carried out a better methodological characterized of these interfaces. They have just briefly stated that the *interfaces were cleaned firmed *[9], or just *cleaned with steel wool *[13]. Neither roughness nor voltage range applied to each EEI have been measured or analyzed.

Consequently, a series of experiments were carried out to solve the controversy to either accept or reject the above authors' statements. In recent years, our group developed a non-linear dielectric spectrometer based on a commercial analyzer, which is expected to solve the EEI interference in tetrapolar cells [26,27]. In order to test the theories of these authors, tripolar and tetrapolar cells were used. The preparation and treatment of the interfaces were carefully carried out to perform the measurements.

Our findings suggest that yeast suspension does not show non-linearities within the applied voltage and frequency ranges; it rather affects the intrinsic EEI behavior. We have monitored changes in the non-linear characteristics of the EEI in presence of yeast with the addition of biochemical activators and inhibitors.

## Methods

### Non-linear spectrometer

**Figure 1:**
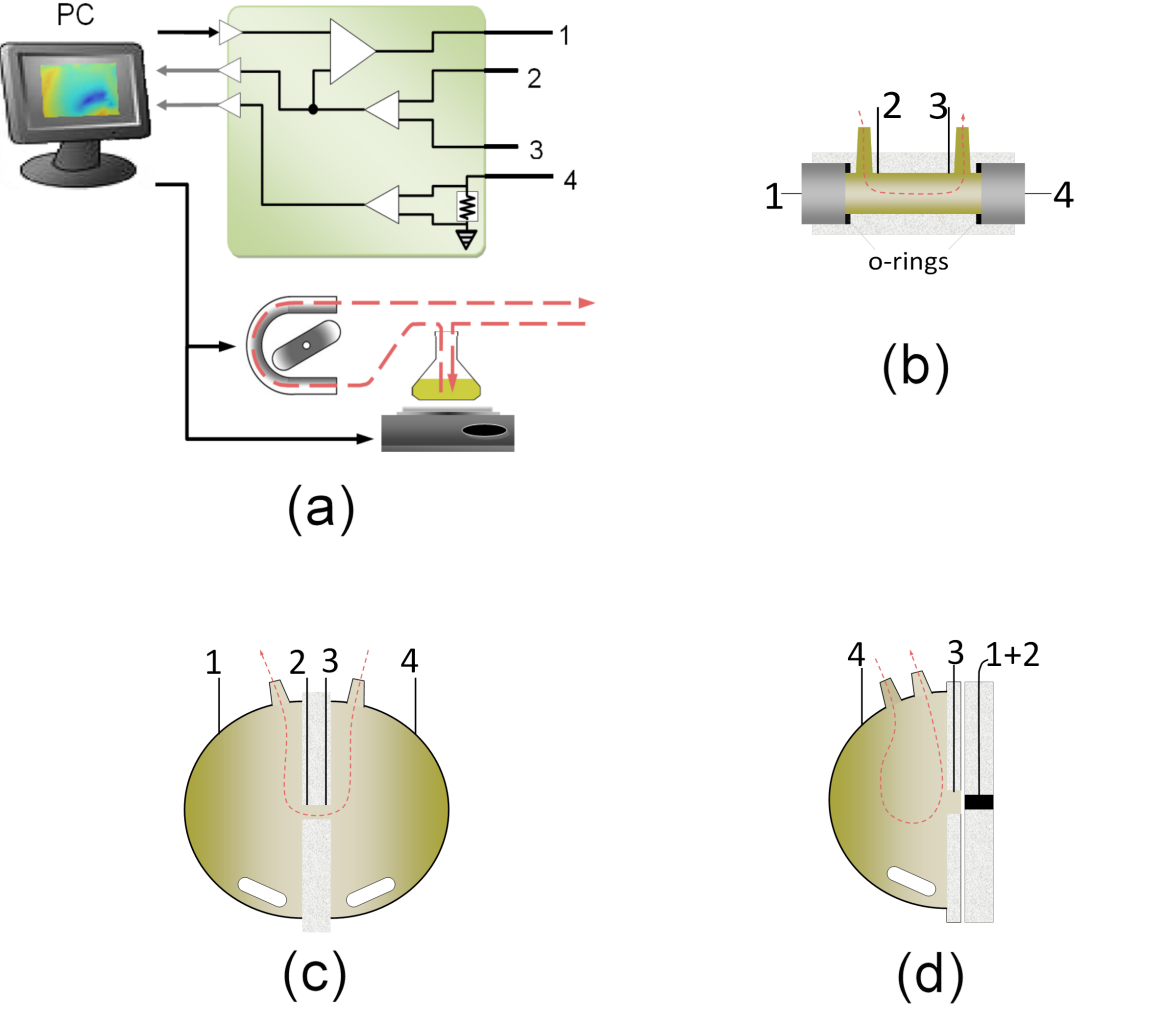
**Non-linear dielectric spectrometer**. (a) Equipment involved, (b) cylindrical flat electrodes tetrapolar cell, (c) low EEI impedance tetrapolar cell, and (d) tripolar cell. In (b) and (c) 1 and 4 correspond to outer electrodes, while 2 and 3 correspond to inner electrodes. In (d) 1 and 2 are connected together and the system acts as a tripolar analyzer, instead of a tetrapolar one. Numbers 1 to 4 correspond to the connections of the SI1287 unit depicted in (a).

The spectrometric system proposed is depicted in fig. 1(a) and consists of a central PC that synchronizes the instruments and collects the data, an electrochemical analyzer Solartron SI1287, a peristaltic pump, a magnetic stirrer and the measurement cell (drawn in subset b-d). The PC is connected to the SI1287 (via serial port), and generates and acquires voltage signals through digital-analog and analog-digital converters (National Instruments).

Details about the operation of the equipment have been published elsewhere [26]. Briefly, the computer generates a sinusoidal voltage signal which is applied through the SI1287 to the biological suspension contained in the cell, by means of two outer electrodes. The voltage is controlled by the SI1287 to provide a sinusoidal (with no harmonics) electric field between the inner electrodes (namely reference electrodes). The current that circulates through the cell is coherently sampled and Fourier transformed by using the periodogram method with a rectangular window [28]. The signal length is chosen to provide, at least, a five samples separation between harmonics.

The frequency estimate is obtained for the entire range, but only the amplitude of the fundamental frequency and its harmonics (2^nd ^to 5^th^) are stored. For each harmonic analyzed, an amplitude grid is computed when both frequency and amplitude of the input signal are changed in discrete intervals. This grid can be used to plot a surface as function of frequency and amplitude of the input signal. All computing and operations are performed by the PC, the SI1287 only serves as electrochemical interface, but does not perform any analysis. All voltages are expressed as RMS values.

The capability of the system was tested with linear and nonlinear phantoms. It was proved that the harmonic content measured in the current signal is strictly dependent on the linear/non-linear system connected between the reference electrodes [27].

The system is completed with a reservoir with solution and a pumping system that recycles the solution through the cell. The stirrer and pump were turned on for 10 seconds after completing 90 seconds of acquisition. Whenever any product was added to the suspension (inhibitors or activator of the H^+^-ATPase) the mixture was stirred and pumped for 1~2 minutes to homogenize the system. Both pump and stirrer are controlled by the PC in order to ensure steady conditions during acquisition.

### Tetrapolar cells

Two different types of tetrapolar cells were developed. One of them (fig. 1(b)) was designed to provide a very uniform electric field within the biological material. It consists in a cylindrical acrylic small volume piece (length 40 mm, diameter 10 mm) with two flat stainless steel outer electrodes (AISI 304). The two inner electrodes are implemented with Dentaurum steel wire (diameter *d *= 1 mm), separated 30 mm in between. The electrodes and cell were designed to let the set be mounted and unmounted before each experiment. The electrodes were polished with metallographic sandpaper grit 600 (average particle size of 14.5 μm, Buehler discs, USA) at a speed of 280 RPM. Two tubing connections are provided to remove bubbles and to recycle the suspension and avoid yeast settling inside the cell.

The other tetrapolar cell (fig. 1(c)) consist of by two outer hemi spherical electrodes, each one with an area of about 150 cm^2 ^and total cell volume of 400 cm^3^. An acrylic piece separates these two chambers, with a small perforation in the center which allows the communication between them. Two additional parallel flat embedded stainless steel electrodes serve as references (Dentaurum steel, *d *= 0.75 mm). The central piece has a hollow with a diameter *d *= 2.75 mm (area ≈ 6 mm^2^) and length *l *= 1 mm. The impedance between reference electrodes whit the cell filled with saline solution (NaCl 0.9%) was 830Ω, which is much greater than the EEI impedance of the outer electrodes. The central acrylic piece can be changed to achieve different dimension of the central hollow, and thus, different medium impedance.

Electric field distribution in each cell was analyzed by means of finite elements. The freely available FEMM software  was used to solve the current (AC) electromagnetic problem. Both metallic and acrylics parts of the cell were drawn, along with the suspension. Each cell was drawn and the mesh spacing was varied according to the cell's geometry. Dielectric parameters (conductivity σ and relative permittivity ε) adopted for the materials are shown in table 1.

**Table 1:** Dielectric parameters used in the FE analysis

**Material**	**Conductivity σ (S/m)**	**Relative permittivity ε**
Yeast suspension	0.65	1000
Acrylic	1 × 10^-9^	3
Gold	2.2 × 10^6^	1
AISI 304	1.3 × 10^6^	1

Yeast conductivity and permittivity were taken from [9] and [38] respectively. Acrylic parameters were taken from the materials library of the FEMM software and both gold and AISI304 were taken from on-line catalogs.

The EEIs were not modeled, and voltage was applied to the boundaries of the injection electrodes in all cells. The field frequency was fixed at 20 Hz, and its magnitude was adjusted to match an estimated 1 V/cm electric field between the reference electrodes. In the longitudinal cells (fig. 1(b) and 1(c)) it was simply calculated as 1 V/cm multiplied by the separation between the outer electrodes. In the spherical cell (fig. 1(d)), the voltage applied to the external electrodes was manually adjusted to match an electric field of 1 V/cm between reference electrodes. Woodward's cell was configured as planar and the other two were modeled as axisymetric relative to the *y*-axis.

### Tripolar Cells

The tripolar cell (fig. 1(d)) has a central working electrode (circular with diameter *d *= 8 mm) bounded in an acrylic disc (external diameter *d *= 100 mm), while a second thin central-hollowed disc (external diameter = 100 mm, diameter of the central hollow *d *= 10 mm) with a stainless steel wire acts as a reference electrode (Dentaurum steel, *d *= 1 mm). The working electrode is hand-polished before each experiment with diamond past and aluminum powder, up to a final roughness of 1 μm. We tested a golden electrode (24 carat) and a stainless steel (AISI 304) working electrode. We have also tested the stainless steel electrode polished with sandpaper under the same conditions of the tetrapolar cell electrodes. The system is completed with a large-area hemi-spherical stainless steel counter electrode. Thus the counter electrode-electrolyte-interface impedance (Z_ce_) is much smaller than the working electrode-electrolyte-interface impedance (Z_we_). The counter electrode is hermetically secured with rubber seals over the acrylic disc, and is provided with tubes to allow the suspension to flow-in and out. A magnetic stirring bar is introduced inside the cell.

In the tripolar cell the voltage is controlled between working and reference electrodes, that is, the voltage drop in the working electrode-electrolyte-interface. The loss in the medium or counter electrode interface is negligible, due to the low medium impedance Z_medium _(Z_Medium _+ Z_ce _« *Z*_we_).

Initially, two opposite tripolar (ideally equal) cells were used, one as reference and the other one as test cell. The responses were subtracted to correct the non-linear behavior of the interface. However, this became almost unpractical because these two interfaces were hardly similar (in their impedance response) even they were made with identical material and equally treated [26]. As an alternative, we decided to use the same interface repeatedly and test the biological response by using an inhibitor or activator of the enzyme, as it was also carried out by Woodward in his latest publications [18,19]. After some reference measurements, the activator was added and some test measurements were performed.

### Experimental procedures

Experiments were initially divided in two groups: bulk or electrode-interface measurements carried out either with tetrapolar or tripolar cells, respectively. In each case all harmonics were analyzed, but statistics and graphics correspond only to the third harmonic because it has been showed to be the most representative of the biological response (see Woodward's and Nawarathna's references).

The tetrapolar experiments were performed by using a single cell and consecutive measurements up to four hours after yeast hydrating. These procedures were divided in two stages. During the first two hours, measurements were carried out on the hydrated yeast, subsequently, a biochemical stimulus was added and measurements were continued for two hours. Measurements before and after the stimulus are used as reference and test, respectively. The stimuli were selected upon the previous references and they are sodium metavanadate (SMV, 1 mM, an inhibitor of the enzyme [29]) and glucose (100 mM, substrate of the enzyme, the activator [30,31]). Both affect the H^+^-ATPase, which was recognized as the source of the non-linear biological response.

A total of 12 experiments were carried for tetrapolar cells with several ranges of electric field. The lowest electric field range tested varied from 0.1 V cm^-1 ^to 1.5 V cm^-1^, whereas the maximum ranged from 7 V cm^-1 ^to 70 V cm^-1^. This high field range was attained with the cell depicted in fig 1(c). The field range was divided in 11 or 21 logarithmic steps. The frequency range was always the same, between 1 Hz and 100 Hz, divided both into 11 or 21 logarithmic steps. Most of previous harmonics generation reports in yeast suspension were accounted in the 1 Hz~100 Hz range.

The amount of measurements depends on the length of both frequency and voltage ranges. When both ranges were built with 11 steps, each complete measurement (11 × 11 single measurement) took about 10 minutes to be completed. These experiments usually covered 10 measurements previous and 10 measurements posterior to the stimuli. When the ranges were divided into 21 steps, each complete measurement took over 40 minutes. In this case, each experiment consisted of 2~3 measurements previous and posterior to the stimuli.

Since electrode corrosion does not influence tetrapolar measurements, and yeast cell count does not significantly vary during the experiments, repeated measurement can be considered independent observations. Tetrapolar data were analyzed with the ANOVA model for repeated measurements with no interaction between factors. Frequency, electric field and type of measurement (reference or test measurement) are treated as independent predictors. Amplitude of the third harmonic, expressed in dB, is used as dependent variable of the model. Values of *p *less than 0.01 are considered significant.

Interface measurements were carried out likewise, with reference and test measurements and stimulus in between. However, as tripolar measurements are influenced by electrode electrochemical corrosion, they were divided in two groups, single and repeated (> 5) measurements prior and after the stimulus. Single measurements are intended to avoid as much as possible electrode corrosion. Consequently, only two measurements are carried out with the stimuli in between. In long term experiments, it is expected to observe a change in the harmonic amplitude coincident with the biochemical stimuli, along with a drift due to electrode corrosion. In such cases, any response should be observed as a time depending process. It was also tested the difference (subtraction) of the average measurement previous and posterior to the stimulus. No statistics analysis was performed over the tripolar measurements, and the data provided is analyzed qualitatively. These experiments were carried out with both gold and stainless steel electrodes with a voltage range of 150 mV~1.5 V between 1 Hz and 100 Hz applied to the working-electrode-electrolyte interface.

### Samples preparation

In order to facilitate comparison with results reported by Woodward, we used the same biological material (e.g. *S. cerevisiae*). The microorganism was obtained locally as a freeze-dried powder, and resuspended up to a concentration of *ca*. 50 mg dry wt ml^-1^, in the same saline solution. All chemicals used were of analytical grade and the water was glass-distilled (final conductivity less than 5 μS cm^-1^). The suspension concentration (10^9 ^CFU ml^-1^) was determined by plate count.

## Results

### Electric field distribution in tetrapolar cells

**Figure 2:**
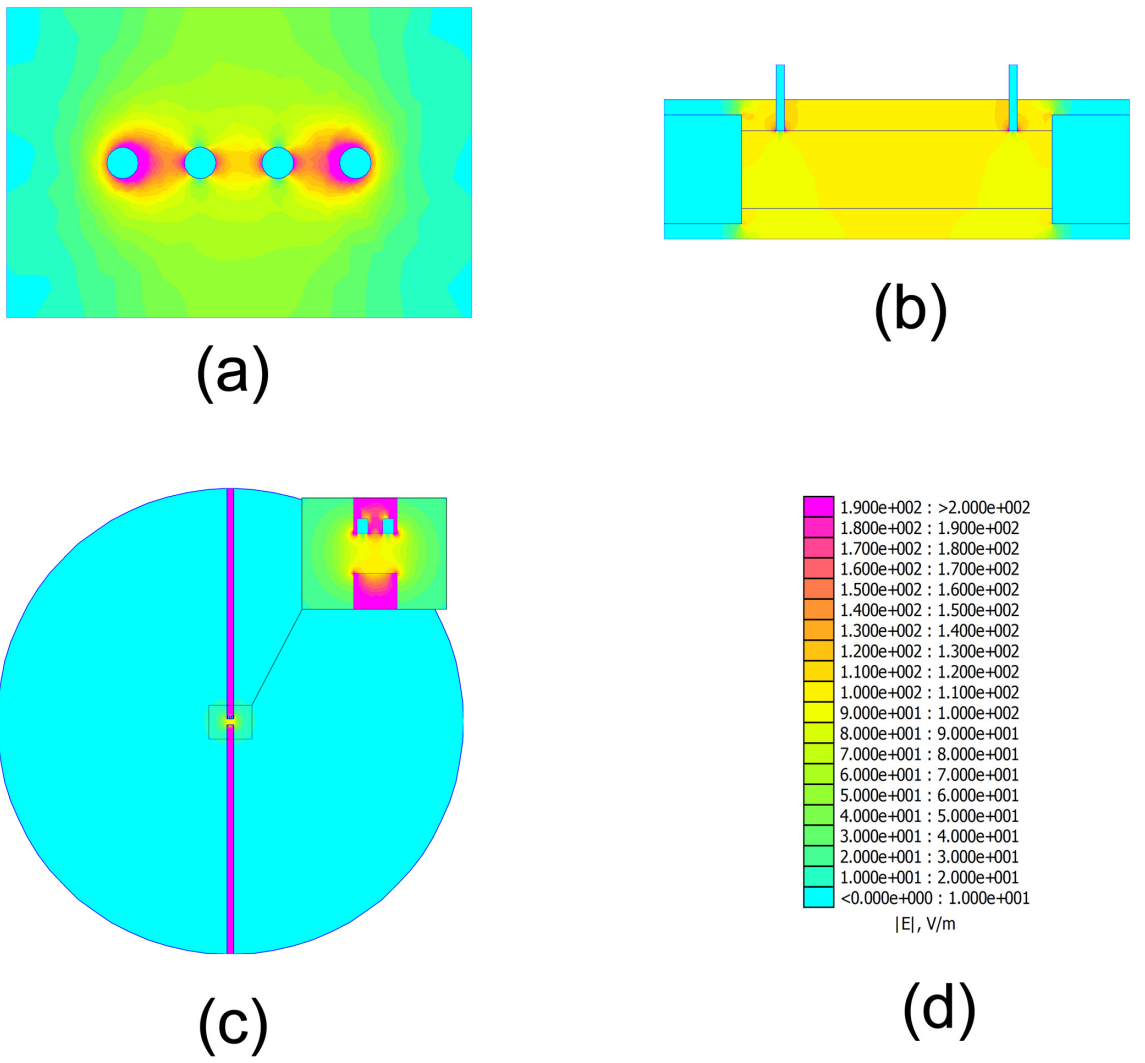
**Electric field distribution in the tetrapolar cells**. Woodward's cell, (b) tetrapolar cell with flat electrodes, and (c) low EEI cell. All figures have been color-coded according to the scale showed in (d). For a better visualization, the field scale was clipped to a maximum of 2 V/cm. However higher values (~3.5 V/cm) were observed close to the outer electrodes in (a) and close to the acrylic corners in (c).

The electric field distribution obtained with the software simulation is presented in fig. 2. The first cell depicted corresponds to Woodward's design (fig. 2(a)). The voltage difference between the boundary of the injection electrodes was 0.75 V (their center are separated by 7.5 mm). The geometry and the field distribution are highly non-uniform in this cell. There are peak values as high as 3 V/cm close to the injection electrodes, and a large peripheral area with much lower values (< 0.5 V/cm). A histogram analysis performed to fig. 2(a) (data not shown) revealed that ~60% of the electric field is minor than 0. 5 V/cm and only 11.3% of the field ranges into the 0.85 V/cm~1.05 V/cm interval.

The analysis of the flat electrodes cylindrical cell (fig. 2(b)) revealed a very uniform electric field but still a field gradient (between 0.2 V/cm and 2 V/cm) was observed in the region close to the inner electrodes. However, 95% of the computed electric field was close to 1 V/cm.

The high volume cell (fig. 2(c)) revealed a wider range of electric field measured in the inner hole. This is mainly due to the gradient generated at the boundaries of the inner hole and also close to the inner electrodes. The field values in this inner hole are normally distributed around the mean value of 1 V/cm.

### Experimental procedures

The first five harmonics were obtained, however, the third harmonic was considered to be the most important and we shall refer to it from now on. We first analyze the measurements performed with tetrapolar cells. Results of the statistical analysis along with some experimental parameters are presented in table 2.

**Table 2:** Results of the tetrapolar analysis

**#**	**Cell**	**Electric field [V/cm]**	**Frequency [Hz]**	**Inhibitor or activator**	**# of measurements before-after**	***p***
1	Cylindrical	0.16~1.33	1~100	MVS	9 -- 10	0.72
2	Cylindrical	0.1~1.5	1~100	MVS	12 -- 18	0.09
3	Cylindrical	0.1~1.5 *	1~100 *	MVS	3 -- 5	0.89
4	Cylindrical	0.16~1.67	5~50	MVS	12 -- 17	0.96
5	Hemispheric	0.5~15	1~100	MVS	9 -- 10	0.38
6	Hemispheric	7~70 *	1~1000 *	MVS	2 -- 2	0.39
7	Hemispheric	0.1~5	1~100	MVS	6 -- 9	0.14
8	Hemispheric	0.5~30	1~100	Glucose	6 -- 5	0.60
9	Hemispheric	0.5~30 *	1~100 *	MVS	5 -- 3	0.65
10	Hemispheric	0.3~30 *	1~100 *	Glucose	3 -- 5	0.28
11	Hemispheric	0.3~30	1~100	Glucose	6 -- 6	0.27
12	Hemispheric	0.3~30	1~100	Glucose	6 -- 7	0.07

The second column indicates the type of cell used, the third and fourth column indicate the electric field and frequency range used to evaluate the suspension (asterisk indicates a 21 points range; otherwise it was divided in 11 points), the fifth column indicates the activator or inhibitor used, the sixth column stands for the number of experiments averaged prior and posterior to the stimuli. The last column shows the *p*-value obtained for the test between measurements before and after the stimuli.

**Figure 3:**
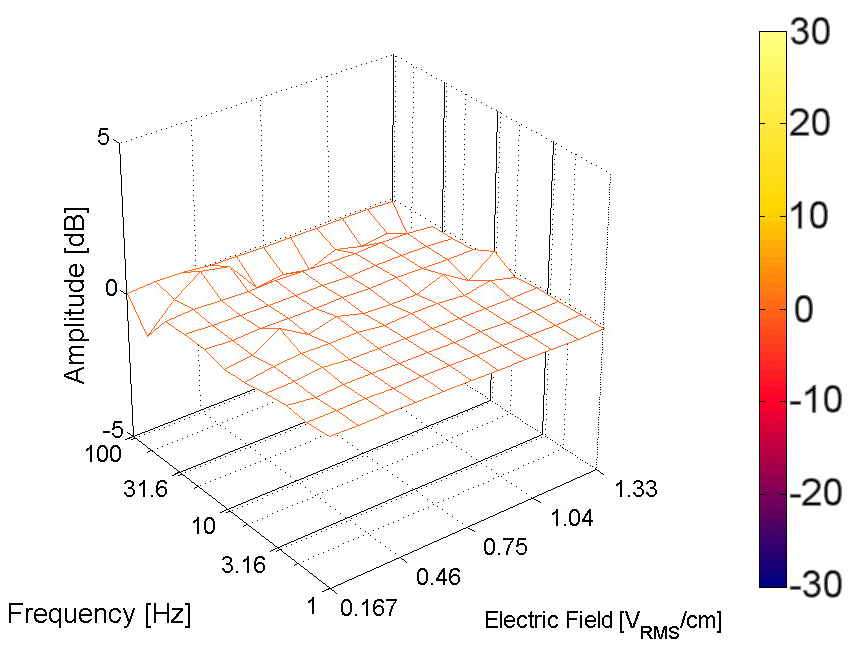
**Difference between averaged measurements before and after addition of SMV in tetrapolar cells**. The horizontal axes stand for the frequency and amplitude of the applied electric field. The vertical axis stands for the amplitude of the third harmonic measured at that combination of frequency and amplitude. The experiment was performed with the flat parallel electrodes cylindrical cell showed in fig. 1(b). Frequency range was 1 Hz~100 Hz (logarithmic scale, 11 steps), and field range was: (a) 0.167 V cm^-1 ^~1.33 V cm^-1 ^(logarithmic scale, 11 steps). Surface is color-coded according to the color bar presented.

In every tetrapolar measurement the relative amplitude of the third harmonic (as expressed to the fundamental amplitude) was lower than -60 dB. Neither electric field nor frequency seemed to affect the harmonic content observed. Furthermore, addition of SMV or glucose did not either modify the harmonics. Fig. 3 shows the difference between averaged measurements previous and posterior to a single application of SMV, which corresponds to the first experiment described in table 2. The algebraic operations were performed upon single surfaces expressed in dB.

The surface obtained is almost flat, with its amplitude very close to 0 dB, with no response attributable to biological source. The same result was obtained when the stimulus was glucose and when other harmonics were analyzed. The same response was obtained in other experiments using even higher voltages (up to 1.67 V cm^-1^). A total of four experiments are reported using the same cell and moderated electric fields, all tested with SMV (row 1 to 4, in table 2).

The low EEI impedance hemispheric tetrapolar cell was then evaluated with even higher electric fields, but the results obtained were almost identical to the former cell. The response was a noisy surface, with its amplitude comprised between -5 dB and 5 dB, but no reproducible biological response was observed. It was also tested the effect of glucose, and also all harmonic were analyzed, and the same results were obtained. All these experiments are detailed in table 2 (rows 5 to 12).

There was no evidence of harmonic generation or biological interaction within the electric field and frequency ranges tested. Statistical analysis revealed no difference between test and reference measurements (*p *> 0.01) for all the tetrapolar experiments performed and this result was independent from the type of stimuli and cell used.

After performing tetrapolar measurements without noticeable results, measurements at the EEI in the tripolar configuration were carried out as showed in fig. 1(d). Earliest experiments were conducted with only two measurements with the stimulus in between, to reduce the electrode corrosion process.

**Figure 4:**
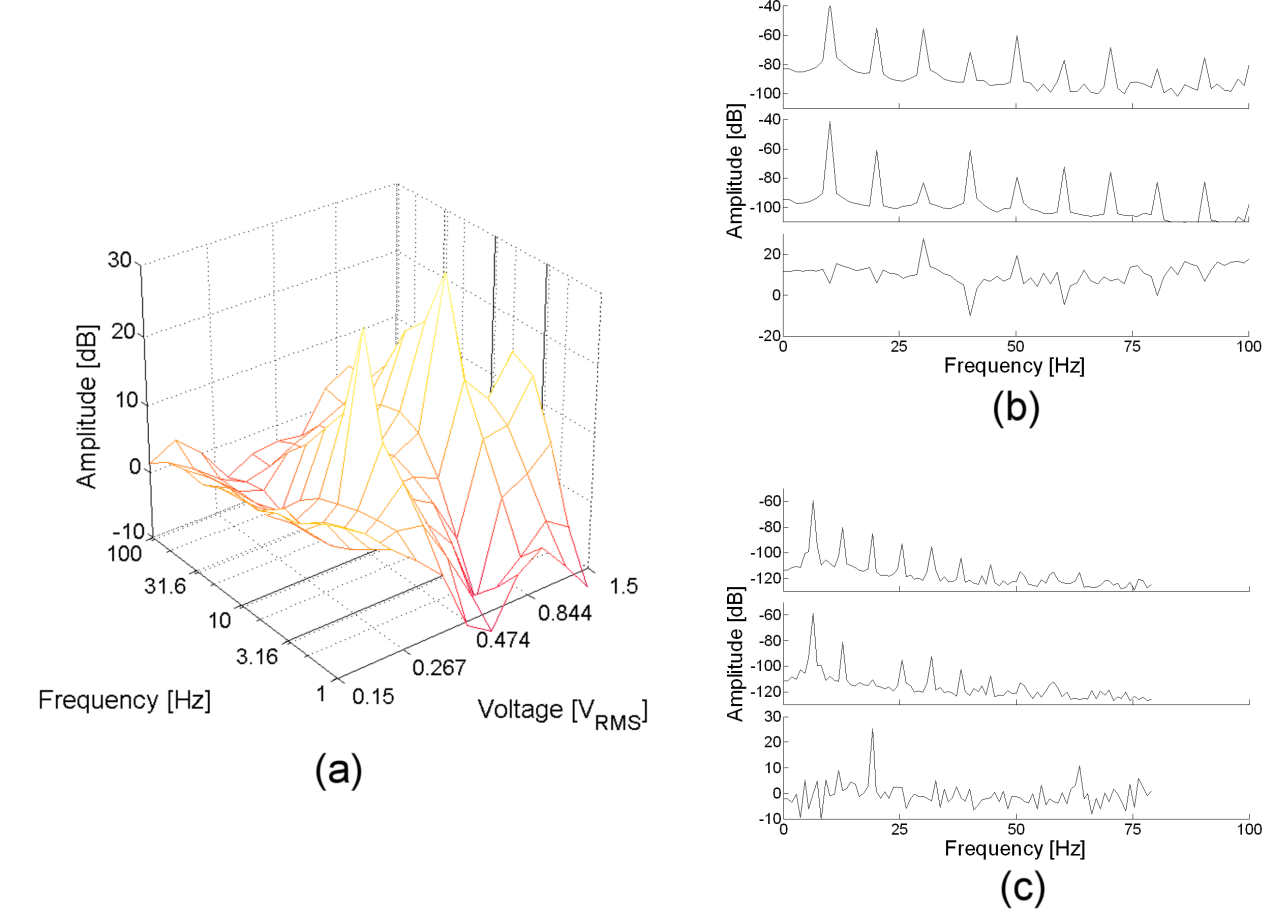
**Third harmonic response measured in tripolar cell with gold electrode**. Subsets (a) show the difference between a single measurement before and after a single injection of SMV. For the two peaks observed, the test, reference and difference spectrums were plotted at the right of the image. Subset (b) shows the spectra when the signal strength was 946 mV at 10 Hz. The difference measured for the third harmonic is 27.4 dB peak. Subset (c) shows the spectra when the signal strength was 376 mV at 6.3 Hz. The difference measured for the third harmonic is 26.3 dB. The surface in (a) is color-coded according to the color bar presented in fig. 2.

When SMV was added, a reproducible change in the third harmonic amplitude was observed, as presented in fig. 4. Subset (a) shows the difference between the measurements before and after the SMV injection. Two distinctive peaks were observed at the surface, and their amplitude was greater than 25 dB. For each peak, the corresponding frequency spectra were plotted at the right of the image (subsets (b) and (c)). From top to bottom, each spectrum corresponds to test (hydrated yeast), reference (hydrated yeast plus SMV) and its difference, respectively. Both peaks are generated because the third harmonic was decreased or abolished after the addition of SMV.

The experiment was repeated, but mostly only one peak was observed. However, in every case the third harmonic was abolished after the addition of SMV, and there is a difference close to 25 dB between the test and reference spectrum. The frequency and voltage values for every peak detected are detailed in table 3.

**Table 3:** Results of tripolar analysis

**#**	**Material**	**Frequency [Hz]**	**Voltage [V_RMS_]**	**Amplitude [dB]**
1	Gold	10.0	0.94	27.4
2	Gold	6.31	0.37	26.3
3	Gold	8.57	0.69	42.4
4	Gold	6.31	0.94	26.3
5	AISI 304/1 μm	6.31	0.94	-20.6
6	AISI 304/papersand 600	3.98	0.94	-25.1

7	Gold	10.0	0.46	14.5
8	Gold	25.1	0.46	10.0
9	Gold/glucose	15.9	0.94	11.8

Each row indicates the appearance of a peak or valley in the third harmonic difference before and after the stimuli. Rows 1 to 6 correspond to a single measurement before and after the stimuli, whereas rows 7 to 9 correspond to repeated measurements. The second column indicates the material of the electrode. The third and fourth column indicates the frequency and voltage where a maximum was observed. The fifth column indicates the amplitude of that maximum. Gold electrode were polished to 1 μm, and stainless steel (experiments 5 and 6) was polished as indicated in column one. All experiments were performed with inhibitor of the enzyme (SMV), except experiment #9 where glucose was added.

**Figure 5:**
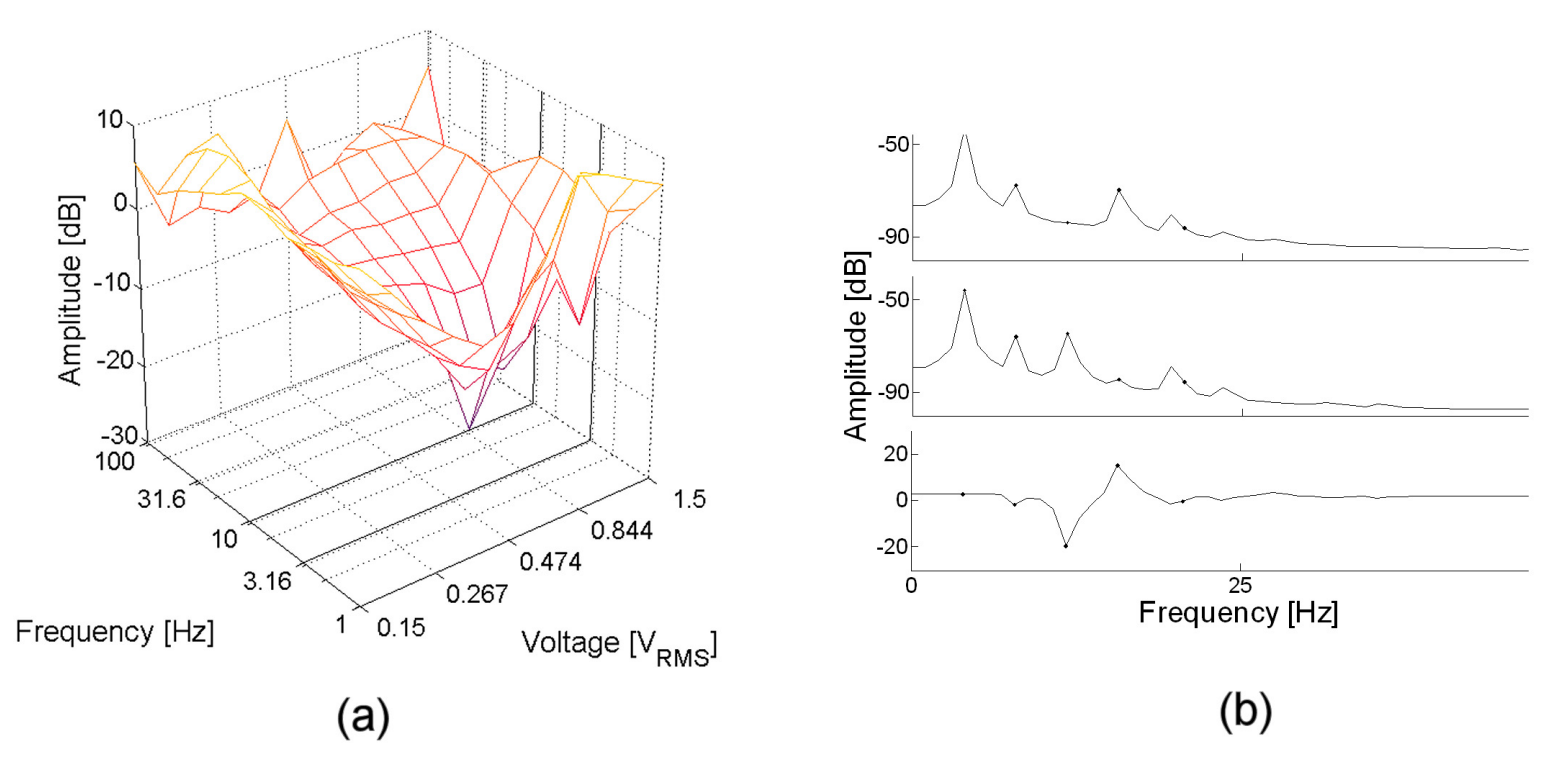
**Third harmonic response measured in tripolar cell with stainless steel electrode**. Subsets (a) show the difference between a single measurement before and after a single injection of SMV. For the valley observed, the test, reference and difference spectrums were plotted at the right of the image, in subset (b). The surface in (a) is color-coded according to the color bar presented in fig. 2.

When using the polished (1 μm diamond past) stainless steel electrode (fig. 5), the surface showed a valley (instead of a peak) for very low frequencies (~4 Hz). However, for this material, the third harmonic was not present in the test spectrum, and it appeared in the reference spectrum. Similar result was observed when the electrode was polished with sandpaper grit 600 (table 3, experiments 5 and 6 respectively).

Thereafter, several measurements were carried out, the stimulus was applied and then measured again. The measurements were performed throughout the entire voltage and frequency range stated in materials and methods. After that, the amplitude measured for a single combination of voltage and frequency was extracted from each surface and plotted against the time of measurement. Time zero corresponds to the time when hydration and mixture of the dried yeast with the saline solution started.

**Figure 6:**
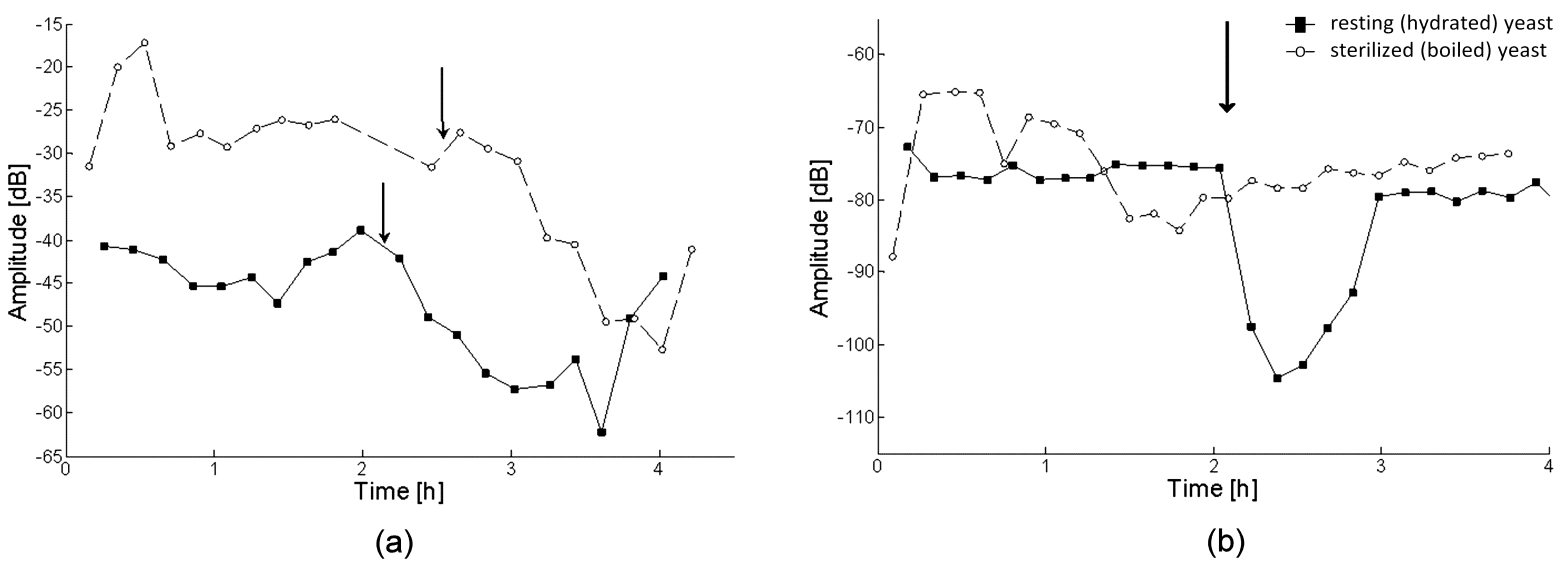
**Time evolution of the third harmonic when measured repeatedly on gold electrode adding SMV and glucose**. (a) two different experiments where 1 mM of SMV was added at the time indicated by the arrow. (b) Effect of glucose on resting and sterilized yeast. Glucose (100 mM) was added at the timeindicated by the arrow in both experiments.

Fig. 6 shows the temporal evolution of the third harmonic in two different experiments. Both lines correspond to a voltage of 460 mV, but the frequencies were 25 Hz (-▮-) and 10 Hz (-◦-) for each experiment. The arrows indicate the time when SMV was added. There is a shift of the harmonic baseline and its amplitude decreased after adding SMV. These two plots corresponds to experiments #7 and #8 of table 3. Subset (b) shows the time evolution of the third harmonic at 946 mV and 15.9 Hz, when glucose (100 mM) was added, as indicated by the arrow. A decrease of 30 dB was observed after ~30 minutes, and it was reestablished one hour after the glucose injection (experiment #9 in table 3).

When measurements were repeated with the stainless steel electrodes, the electrochemical corrosion of the electrode produced a high drift of the baseline, previous to the stimulus injection. We did not observe reproducible responses on steel electrode, despite of the electrode polishing or previous experimental treatment. The magnitude of the drift was close to 20 dB/hour and did not stabilize during the four hours of experiments (data not shown).

## Discussion

Both bulk medium and electrode-interfaces were tested with the same treatment and analysis in order to obtain biological non-linear interaction. In all cases, changes were analyzed up to the fifth harmonic, and the relevant ones were documented here. In order to confirm true bulk non-linear response, tetrapolar cells were first used. No relevant response were found for any harmonics, independently of the cell arrangement (low EEI or flat parallel electrodes), despite that high electric fields (> 10 V cm^-1^) were applied. Statistical analysis confirmed these statements. All measurements showed linear response, and no variations were observed even when SMV or glucose was added.

Repeatable results were found when the analysis was focused on the current through the EEI, while sustaining a sinusoidal voltage. Changes have been observed in the 3^rd ^harmonic when SMV was added, and the spectral changes observed, close to 30 dB, were similar to the previously published. However, the voltage and frequency of appearance of each peak were not the same in all experiments; there was a day-by-day variation in the exact magnitude and location of the 3^rd ^harmonic.

Time evolution changes were observed when both SMV and glucose were added. These changes are also consistent with previous results of other authors [18,23], nevertheless changes did not occur always at the same voltage-frequency values.

Due to the non-linear nature of the EEI, tripolar measurements must be corrected to "subtract" the interface contribution. Consequently, stable interface are still required with this arrangement. The cells were intended to facilitate the polishing procedure, but this did not guarantee repeatable interfaces. We have tested other materials for electrodes, including copper and graphite. The graphite electrode was the most stable one, but no detectable changes were found when the stimulus was applied. We have also tested several polishing degrees for stainless steel electrodes, but it was very unstable and suffered rapid and severe corrosion due to the high voltage applied. The effect of electrode instability and material was extensively analyzed by Woodward and co-workers [18]. Gold electrodes were chosen among several metal to be the most stable one, but the surface treatment of the material had not been emphasized. Our results were highly dependent on the polishing degree, and reproducible results were only observed for polished gold electrodes.

A brief analysis of all instrumentation of cited groups reveals that there are neither two identical spectrometers nor two identical measurement cells. All reported spectrometers used sinusoidal generators applied to outer electrodes of bi and tetrapolar cells, and none of them controlled the waveform of the electric field measured applied to the bulk. This required further correction, as performed by Woodward and Claycomb [9-11,15,16,18,23,24]. Other chose to use a single cell and traced the change in the harmonics amplitude [25,32]. Our design compensated well enough the distortion of the EEI to ensure a true sine electric field applied to the bulk without need for correction. Medium-related harmonics in the voltage signal were sensed and corrected by the driver electrodes, and harmonics were only present in the sensed current.

The cell geometry should also be analyzed as a possible source or harmonics generation. The electric field presented in fig. 2(a) shows how the cell geometry affects the potential measured by the sensing electrodes. The electric field distribution, governed by differential equations, is nothing but homogenous. It seems feasible to observe harmonics in the voltage sensed by the inner electrodes, even if in a true tetrapolar sinusoidal signal was applied to the injection (outer) electrodes. Similar results would be obtained with the pin-type cell used by other groups [23,24,32].

The tetrapolar cells developed and presented in this paper were designed taking into consideration the electric field distribution. The difference between Woodward's results and ours could be related not only to the waveform of the electric field, but also the magnitude and dispersion of the electric field. Even though there is no enough evidence to relate harmonic generation to the cell geometry, the geometry should be considered in future analysis and simulations. On the contrary, it has been pointed out experimentally that linear homogeneous electric fields do not generate harmonics in the sensed current.

Our findings are partially consistent with the hypothesis of Blake-Coleman and co-workers, [13,14] where the interface region is responsible for the generation of harmonics, and it can be altered by the presence of biological cells. It has been proved that cell presence can modify the linear and non-linear properties of the interface region [32-34]. The proposed mechanism of linear interaction, however, must be extended to the non-liner domain to explain these finding. Certainly, the interface width and the electrode roughness are several decades lower than the cell size [17]. The interface will probably be better affected by ionic modifications due to the presence of the yeast cell, rather than a direct modification of the double-layer itself.

Recently, the patch-clamp technique was employed successfully to synchronize and drive the energy conversion of the Na+/K+ ATPase [35-37]. This procedure simplifies the dielectric suspension model (there is a direct measurement of the transmembrane voltage and ionic current). It also resolves the random orientation of the enzymes to the electric field and the membrane surface conductivity of the cell. The information about enzymatic rates, field amplitudes, and signal symmetry should be integrated into a dielectric model of the suspension to provide an equivalent whole-cell equivalent model. This information would contribute to understand the macroscopic dielectric phenomenon of the non-linear interaction.

## Conclusion

The biological phenomenon interaction well reported by many authors (as proved with enzyme inhibitors, variable cell concentration, glucose addition, yeast sterilization and genetic alterations) could not be reproduced in tetrapolar analysis. There was no significant harmonics generation in the bulk suspensions of the organisms tested so far, when applying a pure sinusoidal electric field up to 70 VRMS cm-1.

Our results emphasize the hypothesis that there is no electric distortion in a biological suspension when a strictly sinusoidal electric field is applied. The harmonics observed were due principally to electrode-generated non-linearities which affected the bulk current, and thus modulated the sensed voltage. The cell-induced modulation of the harmonics produced at an electrode-suspension interface may be modified by the microorganism response to inhibition or activation.

## Competing interests

The authors declare that they have no competing interests.

## Authors' contributions

EFT carried out the spectrometer development and the experimental procedures, and drafted the manuscript. EFT and CJF participated in the design of the study and performed the statistical analysis. CJF conceived of the study, and participated in its design and coordination and helped to draft the manuscript. All authors read and approved the final manuscript.
